# The journey from congress to journal: publication patterns of ECR 2019 oral presentations

**DOI:** 10.1186/s13244-025-02198-w

**Published:** 2026-02-16

**Authors:** Ali Salbas, Raşit Eren Büyüktoka, Murat Yogurtcu, Münevver İlke Kaya, Binnur Emre, Ali Murat Koc

**Affiliations:** 1 Department of Radiology, Izmir Katip Celebi University, Ataturk Training and Research Hospital, Izmir, Turkey; 2 Department of Radiology, Izmir City Hospital, Izmir, Turkey; 3 Department of Radiology, Tire State Hospital, Izmir, Turkey

**Keywords:** Bibliometrics, Congresses as topic, Journal impact factor, Publishing, Radiology

## Abstract

**Objectives:**

To evaluate the publication outcomes of oral presentations delivered at the European Congress of Radiology (ECR) 2019 and examine factors influencing conversion to full-text articles; findings were also compared with ECR 2010.

**Materials and methods:**

A total of 1817 oral presentations from ECR 2019 were analyzed. Publication status was determined by searching PubMed/MEDLINE up to December 2023. For each matched article, Journal Impact Factor (JIF) and Google Scholar citations/year were recorded. Additional variables included country of origin, collaboration type, imaging modality, and study design. Statistical analyses used chi-square and Kruskal–Wallis, with *p* < 0.05 considered significant.

**Results:**

Of 1817 oral presentations, 844 (46.5%) were published, with no significant difference from ECR 2010 (43%, *p* = 0.091). Abstracts originated from 71 countries, with Italy (16.5%) and China (15.5%) contributing the most. Publications appeared in 254 journals. Publication rates varied significantly by country (*p* < 0.001), with Switzerland (74.4%) and the Netherlands (68.8%) achieving the highest rates. When analyzed by continent, abstracts from Asia showed a significantly higher publication rate than those from Europe (52.3% vs. 43.6%, *p* = 0.001). Publication outcomes also varied significantly by imaging modality (*p* = 0.002) and subspecialty (*p* < 0.001). Breast imaging achieved the highest median JIF (4.9), whereas Artificial Intelligence/Machine Learning (AI/ML) demonstrated the highest median annual citation count (10.5).

**Conclusions:**

Nearly half of the ECR 2019 oral presentations achieved peer-reviewed publication, maintaining rates from 2010. The congress’s contributor landscape has become more global, with greater participation from Asia. While traditional radiological fields remain prevalent, AI/ML abstracts demonstrated high citation rates. These findings reflect contemporary trends in radiological research.

**Critical relevance statement:**

By analyzing the publication outcomes of ECR 2019, with comparisons to 2010 data, this study examines evolving global patterns in publication outcomes, offering insights to enhance the dissemination of radiological research.

**Key Points:**

- Converting oral presentations to publications remains challenging in radiological research.
- Nearly half of the ECR 2019 oral presentations were published, showing a modest, non-significant increase from ECR 2010.
- The congress has become increasingly global, with notable growth in participation from Asia.
- This study reveals radiology’s evolving scientific landscape and current research priorities.

**Graphical Abstract:**

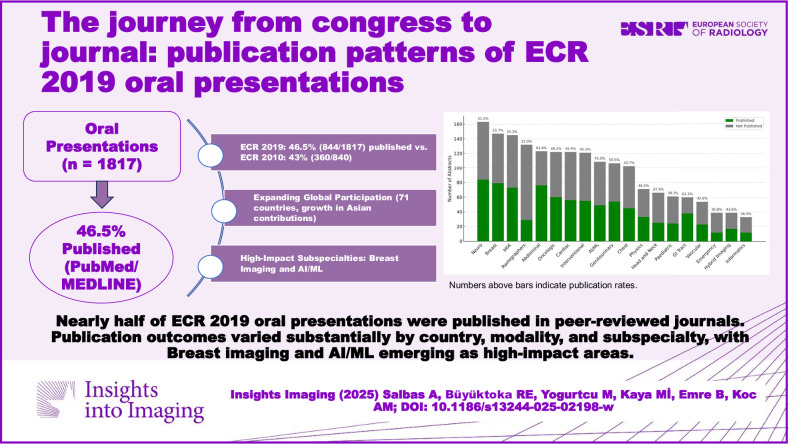

## Introduction

Scientific congresses provide platforms for researchers to share data, exchange knowledge, and foster collaboration. They enable rapid dissemination of findings and improve studies through scholarly critique. Only a portion of congress abstracts are later published as full-text articles [1]. This rate, influenced by various factors, is widely accepted as an indirect indicator of a meeting’s scientific quality [2, 3].

Although many studies have examined this topic in other medical specialties, research on the publication process of radiology abstracts remains limited [1, 4, 5]. The European Congress of Radiology (ECR), held annually in Vienna, is the leading platform in Europe for presenting original radiology research. In 2019, it hosted 30,259 participants from 133 countries, underscoring its international scope [6]. The publication of ECR 2010 oral presentations and influencing factors has been examined in two independent studies [3, 7]. To our knowledge, no subsequent study has evaluated the publication conversion of ECR abstracts or other large-scale international radiology congresses. Radiology is a rapidly evolving discipline shaped by technological advances, making it essential to track publication trends with up-to-date data. This study primarily assessed the publication rate of oral presentations at ECR 2019 converted into PubMed/MEDLINE-indexed articles and examined influencing factors, with comparisons to 2010 findings to provide additional context [3].

## Materials and methods

This study was approved by the Institutional Ethics Committee (Health Research Ethics Committee of Izmir Katip Celebi University; approval number: 2025-SAEK-0732, dated 11/09/2025; Decision no: 0489) and evaluated the publication rate of oral abstracts from ECR 2019 subsequently published as full-text original research articles indexed in PubMed/MEDLINE. The design and methodology were planned to be consistent with the study of Loughborough et al, which analyzed oral presentations from ECR 2010, to ensure comparability [3]. Because ECR 2020 was held online due to the COVID-19 pandemic, we selected ECR 2019 as the last in-person congress with an adequate follow-up period to allow conversion of abstracts into full-text publications [6]. A uniform follow-up duration of 4 years and 9 months after the congress was applied, identical to the follow-up period used in the ECR 2010 analysis, to ensure methodological consistency and allow valid comparison between the two cohorts. More recent ECR meetings (2022–2025) were not analyzed due to insufficient follow-up time.

Abstracts from ECR 2019 were reviewed using the ‘ECR 2019 Book of Abstracts,’ published as a supplement to *Insights into Imaging* [8]. Of 1853 abstracts screened, 36 already published as full-text articles before March 2019 were excluded [9], leaving 1817 oral presentations. Four researchers retrospectively evaluated the presentations. For each abstract, the first author’s city, country, and continent were recorded. For the purposes of regional classification, “Europe” was defined to include the 28 member states of the European Union as of 2019 and Bosnia and Herzegovina, Belarus, North Macedonia, Moldova, Russia, Norway, Switzerland, Turkey, and Ukraine [10].

Because the ECR 2019 Book of Abstracts did not provide detailed institutional affiliations, a modified classification system based on geographical location was used to assess collaboration. Co-authorship was categorized as local (all authors from the same city), national (authors from different cities within the same country), or international (authors from institutions in at least two different countries).

Imaging modalities were classified as CT, MRI, Ultrasound, Fluoroscopy, Radiography, Mammography, PET-CT, non-PET nuclear medicine, Multimodality, or Other. The ‘Other’ category included abstracts without a defined modality, such as survey-based studies. Unlike ECR 2010 [3], a separate ‘Multimodality’ category was added for studies using two or more modalities (e.g., combined CT and MRI), reflecting their increasing use in radiological research. Study design was categorized as retrospective, prospective, or other (e.g., experimental, review, survey-based) [11].

To determine publication status, MEDLINE was searched via PubMed [12]. Before data collection, all researchers were briefed on the review process, including predefined matching criteria and classifications. Searches began with the first author’s name; if no match was found, other authors or title keywords were used [3]. Matches were confirmed by three researchers based on author names, title similarity, topic, and methodology; disagreements were resolved with a fourth reviewer. The follow-up was 4 years and 9 months, consistent with the previous study, including articles published between March 2019 and December 2023. Only full-text original research articles indexed in MEDLINE were included, while posters, case reports, reviews, editorials, and letters were excluded.

For each published abstract, the journal’s impact factor (JIF) was obtained from Journal Citation Reports for the publication year [13]. Data were recorded by one researcher and verified by another. Articles were ranked by JIF, and a “high-impact group” was defined as those published in journals with JIF > 5.0, comprising 210 articles across 61 journals [13].

As an additional step not included in the previous study, the publication year of each article and the total number of citations (as of September 2025) were also recorded using Google Scholar [11]. Google Scholar was preferred for citation tracking due to its comprehensive coverage of all publication types and its accessibility without institutional restrictions. To account for differences in publication year, total citation counts were normalized by dividing them by the number of years since publication, yielding citations per year (citations/year).

### Statistical analysis

Descriptive statistics were reported as frequencies and percentages for categorical variables and as mean ± standard deviation, median, interquartile range (IQR), and minimum–maximum for continuous variables. Publication rate differences between ECR 2010 and 2019 were assessed with the chi-square test. The association between country of origin and publication rates was also tested by chi-square, restricted to countries with ≥ 10 published articles. Normality of continuous variables was assessed with the Shapiro–Wilk test. Associations between categorical variables (e.g., study design, collaboration type) and publication status were examined with chi-square, while continuous variables (e.g., JIF, citations per year) were compared with the Kruskal–Wallis test. Effect sizes were calculated to quantify the magnitude of significant associations: Cramér’s V for chi-square tests and epsilon squared (ε²) for Kruskal–Wallis tests. A *p*-value < 0.05 was considered significant. Analyses were performed using IBM SPSS Statistics for Windows, Version 26.0 (IBM Corp.).

## Results

**Fig. 1:**
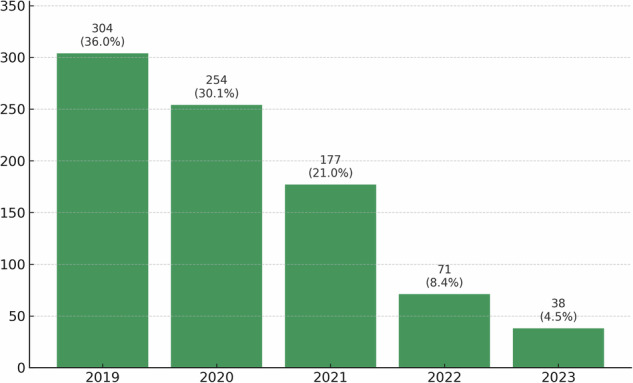
Distribution of publication years for abstracts presented at ECR 2019

A total of 1817 oral presentations were identified at ECR 2019, of which 844 (46.5%) were published as full-text articles indexed in PubMed/MEDLINE. There was no significant difference in publication percentage compared to ECR 2010 (ECR 2019: 844/1817 vs. ECR 2010: 360/840, *p* = 0.091). Both congress years were evaluated using the same follow-up period of 4 years and 9 months. The median JIF was 3.7 (IQR: 2.7–5.2; range 0.3–158.5). Publications received a median of 18.0 citations (IQR: 9.0–33.0; range 0–664). Publication occurred most frequently in 2019 (*n* = 304, 36.0%) and 2020 (*n* = 254, 30.1%) (Fig. 1).

Oral abstracts at ECR 2019 came from 71 countries. The top contributors were Italy (*n* = 299, 16.5%), China (*n* = 281, 15.5%), and Germany (*n* = 244, 13.4%).

A total of 51 countries subsequently published at least one abstract as a full-text article, while no publications were identified from 20 countries. Germany had the highest number of publications (*n* = 157, 64.3%). A total of 14 countries had ≥ 10 publications (Table 1). Publication rates differed significantly between countries (χ² = 87.8, *p* < 0.001, Cramér’s V = 0.25). Among them, the highest publication rates were observed in Switzerland (74.4%) and the Netherlands (68.8%). The Netherlands additionally had the highest percentage of articles published in high-impact journals (40.0%) (Table 1).

**Table 1:** European Congress of Radiology (ECR) 2019 publication rates, JIF, and citations per year according to country of origin

Countries	Abstracts	Published	High-impact journals	JIF	Citations/year
Italy	299 (16.5)	136 (45.5)	31 (22.8)	3.5 (2.7–4.9)	3.8 (2.0–6.9)
China	281 (15.5)	131 (46.6)	25 (19.1)	4.0 (2.8–4.7)	4.3 (2.8–8.7)
Germany	244 (13.4)	157 (64.3)	45 (28.7)	4.1 (3.0–5.3)	4.0 (2.2–6.6)
Netherlands	80 (4.4)	55 (68.8)	22 (40)	4.6 (3.1–5.4)	3.3 (2.2–6.8)
United States	78 (4.3)	31 (39.7)	9 (29)	4.0 (3.0–6.4)	5.2 (2.7–14.2)
United Kingdom	77 (4.2)	28 (36.4)	10 (35.7)	3.7 (2.4–7.0)	4.2 (2.6–7.0)
India	74 (4.1)	15 (20.3)	1 (6.7)	2.3 (1.2–3.6)	2.6 (1.4–3.5)
South Korea	74 (4.1)	46 (62.2)	12 (26.1)	3.5 (3.0–5.2)	3.6 (2.0–6.3)
France	44 (2.4)	21 (47.7)	8 (38.1)	4.0 (2.9–7.0)	3.5 (1.4–12.5)
Switzerland	43 (2.4)	32 (74.4)	12 (37.5)	4.0 (2.9–6.0)	4.8 (2.2–7.6)
Japan	43 (2.4)	18 (41.9)	3 (16.7)	2.8 (2.5–4.5)	3.9 (0.8–5.3)
Austria	37 (2.0)	22 (59.5)	8 (36.4)	4.8 (3.4–6.6)	4.4 (3.1–8.5)
Turkey	31 (1.7)	18 (58.1)	1 (5.6)	3.1 (2.0–3.8)	2.7 (1.4–4.2)
Spain	24 (1.3)	11 (45.8)	0 (0)	2.7 (2.0–4.0)	2.0 (1.8–4.3)
Others (≤ 10)	388 (21.3)	123 (54.9)	23 (18.7)	2.8 (2.1–4.6)	2.5 (1.0–4.6)
Total	1817 (100)	844 (46.5)	210 (25)	3.7 (2.7–5.2)	3.7 (2.0–6.8)

Values in the ‘Abstracts,’ ‘Published,’ and ‘High-impact journals’ columns are presented as *n* (%). Percentages in the ‘Published’ column represent publication rates. High-impact journals show the number and percentage of publications in journals with an impact factor > 5.0. Of the 844 published articles, JIF data were available for 784 publications; median JIF values with interquartile ranges (Q1–Q3) were calculated based on these. Citations per year were calculated as total citations divided by the number of years since publication, and are presented as median (Q1–Q3). The “Others” category includes countries with ≤ 10 published articles. Overall, 71 countries presented at ECR 2019; 14 had > 10 published articles and are listed individually, while the remaining 57 are grouped as “Others.” Statistical comparisons: Publication rates differed significantly between countries (χ² = 87.8, *p* < 0.001). JIF values (*p* < 0.001) and citations/year (*p* = 0.013) also showed significant differences across countries

*JIF* journal impact factor

Among countries with < 10 publications, Denmark also showed a high rate (72.7%, 8/11). Countries with only one abstract, such as Uganda, Slovakia, and Kazakhstan, achieved 100% publication.

JIF values differed significantly between countries (H = 35.4, *p* < 0.001, ε² = 0.05), as did citations per year (H = 26.8, *p* = 0.013, ε² = 0.04). The highest median JIF was in Austria (4.8), while the United States had the highest median citations per year (5.2) (Table 1).

Examining continental distribution, the majority of abstracts came from Europe (*n* = 1109, 61.0%) and Asia (*n* = 545, 30.0%), with the remaining 163 (9.0%) from other continents. Publication rates differed significantly between these two major regions, with abstracts from Asia achieving a higher rate than those from Europe (285/545, 52.3% vs. 484/1109, 43.6%; χ² = 10.65, *p* = 0.001, Cramér’s V = 0.08).

A total of 844 publications were identified, which were distributed across 254 different journals. The most frequent journals were *European Radiology* (*n* = 103, 12.2%) and *European Journal of Radiology* (*n* = 45, 5.4%) (Table 2).

**Table 2:** Most frequent journals for publications derived from ECR 2019 abstracts

Journal	*n*	%
*European Radiology*	103	12.2
*European Journal of Radiology*	45	5.4
*Radiology*	28	3.3
*Scientific Reports*	27	3.2
*British Journal of Radiology*	21	2.5
*Clinical Radiology*	18	2.1
*Radiologia Medica*	18	2.1
*Abdominal Radiology*	17	2.0
*PLoS ONE*	15	1.8
*Acta Radiologica*	15	1.8
*American Journal of Roentgenology*	15	1.8
*Journal of Magnetic Resonance Imaging*	13	1.5
*Investigative Radiology*	13	1.5
*Korean Journal of Radiology*	11	1.3
*American Journal of Neuroradiology*	11	1.3
*European Radiology Experimental*	10	1.2
*Cancer Imaging*	10	1.2
*Cardiovascular and Interventional Radiology*	9	1.1
*Neuroradiology*	9	1.1
*Skeletal Radiology*	9	1.1
Others (< 9)	427	50.5
Total	844	100

Only journals with ≥ 9 publications are listed individually; all others were grouped as “Others.” The “Others” category consisted of 427 publications distributed across 234 different journals

When analyzed according to collaboration type, the highest publication rate was observed in the international collaboration group (51.7%), followed by national (48.5%) and local (44.5%) (Table 3). However, no statistically significant differences were found between collaboration types in publication rates (*p* = 0.067), JIF values (*p* = 0.321), or citations per year (*p* = 0.450).

**Table 3:** Publication rates, journal impact, and citations according to imaging modality, collaboration type, and study design

	Abstracts	Published	High-impact journals	JIF	Citations/year
Modality					
CT	561 (30.9)	244 (43.5)	58 (23.8)	3.7 (2.7–5.1)	3.7 (2.1–7.5)
Fluoroscopy	69 (3.8)	30 (43.5)	2 (6.7)	3.1 (2.5–4.1)	2.6 (1.3–3.6)
MRI	676 (37.2)	352 (52.1)	91 (25.9)	4.0 (2.7–5.2)	3.7 (2.0–6.8)
MMG	81 (4.5)	32 (39.5)	16 (50.0)	5.2 (3.8–7.1)	3.1 (1.9–6.7)
Multimodality	165 (9.1)	73 (44.2)	18 (24.7)	3.6 (2.7–5.1)	4.5 (3.0–6.0)
Nuclear medicine	10 (0.6)	3 (30.0)	1 (33.3)	2.7 (2.4–5.3)	4.7 (3.1–6.3)
Other	33 (1.8)	13 (39.4)	3 (23.1)	3.7 (3.3–4.9)	4.8 (3.0–7.5)
PET-CT	30 (1.7)	14 (46.7)	7 (50.0)	6.6 (2.6–7.0)	5.1 (2.7–8.6)
Radiography	59 (3.2)	15 (25.4)	2 (13.3)	2.6 (2.4–3.1)	2.2 (1.2–3.4)
Ultrasound	133 (7.3)	68 (51.1)	12 (17.6)	3.5 (2.5–5.0)	3.3 (1.8–5.5)
Collaboration					
Local	1131 (62.2)	503 (44.5)	115 (22.9)	3.7 (2.7–5.0)	3.7 (2.0–6.3)
National	423 (23.3)	205 (48.5)	56 (27.3)	3.5 (2.7–5.3)	3.6 (1.8–6.8)
International	263 (14.5)	136 (51.7)	39 (28.7)	3.8 (3.0–5.3)	3.8 (2.2–7.0)
Study design					
Retrospective	975 (53.7)	453 (46.5)	106 (23.4)	3.6 (2.7–5.0)	3.8 (2.0–7.0)
Prospective	701 (38.6)	332 (47.4)	90 (27.1)	3.8 (2.7–5.3)	3.5 (2.0–6.2)
Other	141 (7.8)	59 (41.8)	14 (23.7)	3.6 (2.7–5.2)	3.8 (2.2–9.4)

Values in the ‘Abstracts,’ ‘Published,’ and ‘High-impact journals’ columns are presented as *n* (%). Percentages in the ‘Published’ column represent publication rates. Values in the “High-impact journals” column indicate the proportion of published articles in journals with an impact factor greater than 5.0. Journal impact factor (JIF), presented as median (Q1–Q3). Citations/year were calculated as total citations divided by the number of years since publication and are presented as median (Q1–Q3). The “Other” category included abstracts without a defined study design or imaging modality (e.g., survey-based studies). Nuclear medicine refers to non-PET nuclear medicine studies

Statistical comparisons: For modality, significant differences were observed in publication rates (*p* = 0.002), JIF (*p* = 0.004), and citations/year (*p* = 0.021). For collaboration type, no significant differences were found in publication rates (*p* = 0.067), JIF (*p* = 0.321), or citations/year (*p* = 0.450). For study design, no significant differences were observed in publication rates (*p* = 0.488), JIF (*p* = 0.337), or citations/year (*p* = 0.329)

*CT* computed tomography, *MRI* magnetic resonance imaging, *MMG* mammography, *PET-CT* positron emission tomography–computed tomography, *JIF* journal impact factor

In terms of study design, 975 abstracts (53.7%) were retrospective and 701 (38.6%) were prospective (Table 3). However, no statistically significant differences were observed between study designs in publication rates (*p* = 0.488), JIF values (*p* = 0.337), or citations per year (*p* = 0.329).

Across imaging modalities, the largest number of abstracts was presented in the MRI category, followed by CT (Table 3). The highest publication rates were observed in MRI (52.1%) and Ultrasound (51.1%). Mammography abstracts had a publication rate of 39.5%, and PET-CT abstracts had a rate of 46.7%; in both modalities, 50.0% of the published articles appeared in high-impact journals (Table 3). Regarding journal impact, the highest median JIF values were observed in PET-CT (6.6) and Mammography (5.2). PET-CT demonstrated the highest median citations per year among all modalities (Table 3). Statistically significant differences were observed across modalities in terms of publication rates (χ² = 26.10, *p* = 0.002, Cramér’s V = 0.12), JIF values (H = 24.1, *p* = 0.004, ε² = 0.03), and annual citation counts (H = 19.6, *p* = 0.021, ε² = 0.02).

**Fig. 2:**
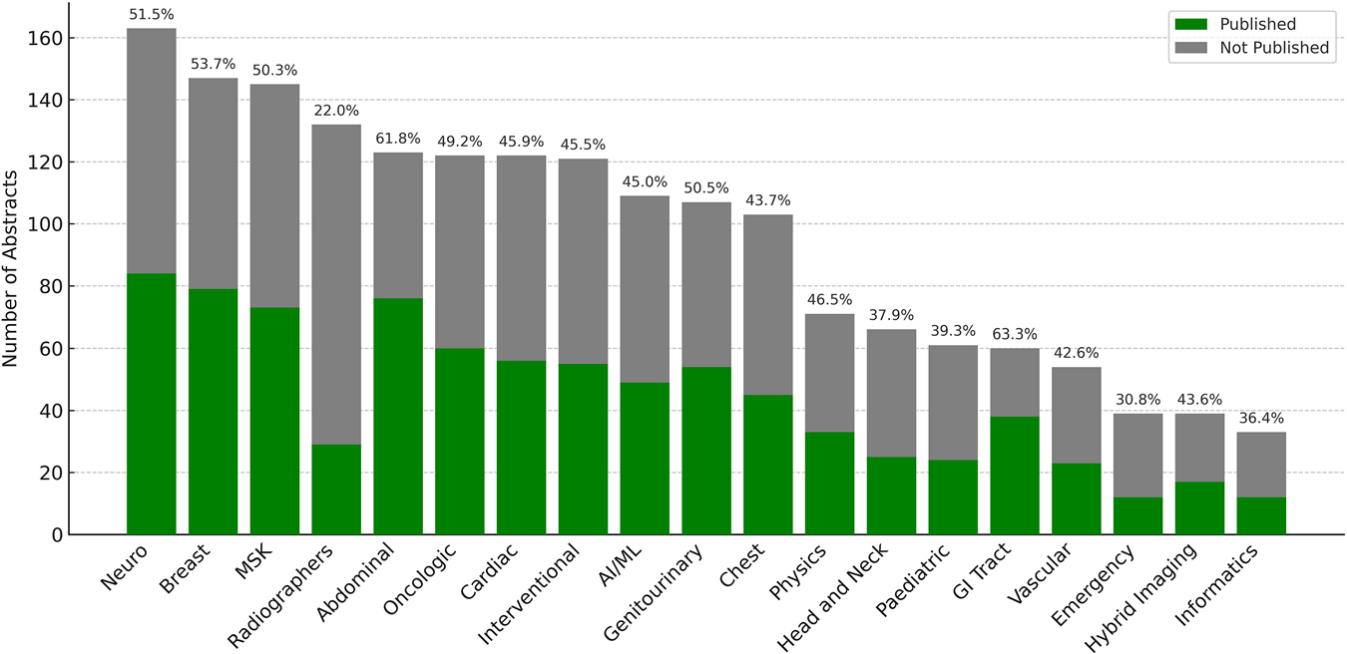
Distribution of abstracts and subsequent publication outcomes across subspecialties. Each bar represents the total number of abstracts presented at ECR 2019, subdivided into published (green) and not published (gray). Percentages above the bars indicate the proportion of abstracts that were subsequently published. AI/ML, artificial intelligence and machine learning

By subspecialty, the largest number of abstracts was presented in Neuroradiology (*n* = 163, 9.0%) (Fig. 2). The highest publication rates were observed in GI Tract (63.3%) and Abdominal Radiology (61.8%) (Table 4). Breast imaging had the highest median JIF (4.9), whereas Artificial Intelligence/Machine Learning (AI/ML) demonstrated the highest median annual citation count (10.5). Statistically significant differences were observed across subspecialties in terms of publication rates (χ² = 66.4, *p* < 0.001, Cramér’s V = 0.19), JIF values (H = 60.6, *p* < 0.001, ε² = 0.08), and annual citation counts (H = 82.4, *p* < 0.001, ε² = 0.10).

**Table 4:** Distribution of publication rates, journal impact, and citations across subspecialties

Subspeciality	Abstracts	Published	High-impact journals	JIF	Citations/year
Neuro	163 (9)	84 (51.5)	25 (29.8)	4.2 (2.9–5.3)	3.6 (1.7–5.6)
Breast	147 (8.1)	79 (53.7)	36 (45.6)	4.9 (3.5–6.4)	3.8 (2.0–7.4)
MSK	145 (8)	73 (50.3)	15 (20.5)	3.3 (2.4–4.5)	3.3 (1.8–4.8)
Radiographers	132 (7.3)	29 (22)	1 (3.4)	2.5 (1.3–3.0)	2.3 (1.0–3.7)
Abdominal	123 (6.8)	76 (61.8)	19 (25)	3.3 (2.7–5.0)	4.0 (2.4–7.0)
Oncologic	122 (6.7)	60 (49.2)	21 (35)	4.1 (3.1–5.9)	4.0 (2.3–7.3)
Cardiac	122 (6.7)	56 (45.9)	16 (28.6)	3.9 (2.9–5.2)	3.6 (1.7–6.0)
Interventional	121 (6.7)	55 (45.5)	5 (9.1)	3.2 (2.7–3.8)	3.0 (1.5–4.4)
AI/ML	109 (6)	49 (45)	11 (22.4)	4.1 (3.6–5.0)	10.5 (5.3–16.0)
Genitourinary	107 (5.9)	54 (50.5)	10 (18.5)	3.0 (2.1–4.3)	4.1 (2.6–6.2)
Chest	103 (5.7)	45 (43.7)	9 (20.0)	4.0 (3.0–4.6)	2.8 (1.8–5.3)
Physics	71 (3.9)	33 (46.5)	7 (21.2)	3.6 (2.7–4.5)	4.5 (1.2–8.3)
Head and neck	66 (3.6)	25 (37.9)	6 (24)	3.6 (1.9–5.5)	3.8 (2.8–5.8)
Pediatric	61 (3.4)	24 (39.3)	5 (20.8)	3.0 (2.5–5.0)	3.3 (2.2–4.8)
GI tract	60 (3.3)	38 (63.3)	8 (21.1)	3.5 (2.4–4.5)	3.8 (2.4–6.5)
Vascular	54 (3)	23 (42.6)	4 (17.4)	3.8 (2.7–5.0)	3.2 (2.1–5.3)
Emergency	39 (2.1)	12 (30.8)	2 (16.7)	4.1 (2.8–5.0)	3.0 (2.5–4.4)
Hybrid imaging	39 (2.1)	17 (43.6)	7 (41.2)	4.1 (3.5–7.2)	6.0 (3.5–15.3)
Imaging informatics	33 (1.8)	12 (36.4)	3 (25)	3.7 (3.1–5.1)	7.4 (4.8–10.8)

Values in the ‘Abstracts,’ ‘Published,’ and ‘High-impact journals’ columns are presented as n (%). Percentages in the ‘Published’ column represent publication rates. JIF (Journal Impact Factor) and citations per year are presented as median (Q1–Q3)

Statistical comparisons: Publication rates (χ² = 66.4, *p* < 0.001), JIF values (*p* < 0.001), and citations/year (*p* < 0.001) differed significantly across subspecialties

*AI/ML* artificial intelligence and machine learning, *MSK* musculoskeletal imaging, *GI tract*, gastrointestinal tract imaging

## Discussion

In this study, we analyzed the publication outcomes of 1817 oral presentations delivered at ECR 2019. Overall, 46.5% were subsequently published as full-text articles indexed in PubMed/MEDLINE. Publication patterns varied significantly across countries, imaging modalities, and subspecialties.

When compared with the analysis of oral presentations from ECR 2010, which reported a publication rate of 43%, the proportion observed in our study (46.5%) showed a slight increase, although this difference did not reach statistical significance [3]. This finding suggests that publication rates have remained relatively stable over time, as the two congress years showed comparable outcomes using the same follow-up period and methodology. However, another investigation of ECR 2010 reported a higher publication rate of 51.8%, a discrepancy that may be explained by differences in the follow-up period and other methodological factors [7].

Two meta-analyses examining publication outcomes across various medical specialties have reported overall conversion rates in a similar range. A Cochrane review including 307,028 congress abstracts estimated a pooled publication rate of 37%, while another meta-analysis of 28 studies calculated a weighted mean publication rate of 41.8%, with values ranging between 3.8% and 78.0% [1, 14]. Although publication rates in radiology and other medical fields are influenced by methodological differences such as the databases searched and the duration of follow-up, the 46.5% publication rate observed in our analysis may be considered moderate to moderately high compared with these broader estimates (Table 5) [3, 7, 11, 15–23].

**Table 5:** Comparison of publication rates across international radiology and other medical meetings

Congress	Specialty	Publication rate (%)	Database(s) searched	Follow-up period
ECR (2000)	Radiology	47.0	PubMed	4 years 9 months
ECR (2001)	Radiology	45.0	PubMed	4 years 9 months
ECR (2010)	Radiology	43.0	PubMed	4 years 9 months
ECR (2010)	Radiology	51.8	PubMed	5 years
**ECR (2019, current study)**	Radiology	46.5	PubMed	4 years 9 months
RSNA (1995)	Radiology	33.0	PubMed	5 years
ESPR (2013–2015), SPR (2013–2015), IPR (2016)	Paediatric radiology	46	PubMed	5–7 years
CIRSE (2012)	Interventional radiology	46.8	PubMed + Google Scholar	3 years
SIR (2012)	Interventional radiology	44.1	PubMed + Google Scholar	3 years
SSR (2010–2015)	MSK radiology	50.6	PubMed + Google Scholar + Google	2–7 years
ESGAR (2000–2001)	GI radiology	39.5	PubMed	3–4 years
AAOS (2016–2017)	Orthopedics	71.6	PubMed + Google Scholar	4–5 years
AAO (2008)	Ophthalmology	57.8	PubMed	6 years
ASA (2009)	Anesthesiology	22.0	PubMed + Google Scholar	~3 years
NASS (2009–2011)	Spine surgery	51.0	PubMed	3–5 years

Sources: Data compiled from the cited references [3, 7, 11, 15–23]

*AAO* American Academy of Ophthalmology, *AAOS* American Academy of Orthopaedic Surgeons, *ASA* American Society of Anesthesiologists, *CIRSE* Cardiovascular and Interventional Radiological Society of Europe, *ECR* European Congress of Radiology, *ESGAR* European Society of Gastrointestinal and Abdominal Radiology, *ESPR* European Society of Paediatric Radiology, *IPR* International Pediatric Radiology, *NASS* North American Spine Society, *RSNA* Radiological Society of North America, *SIR* Society of Interventional Radiology, *SPR* Society for Pediatric Radiology, *SSR* Skeletal Society of Radiology, *MSK* musculoskeletal imaging

Previous studies have shown that the majority (80–94%) of congress abstracts are published within the first 3 years following presentation [11, 16]. In our analysis, a similar pattern was observed, as 87.1% of the subsequently published abstracts appeared in journals within 3 years of ECR 2019.

Our analysis of presentations by country of origin reveals both long-standing trends and significant new developments in the ECR’s landscape. Previous studies of ECR 2000 and 2010 reported that abstracts from several European nations, such as Switzerland, the Netherlands, and Germany, had high conversion rates into full-text articles [3, 7, 9]. Our findings from ECR 2019 indicate that this pattern has continued, with Switzerland (74.4%) and the Netherlands (68.8%) again showing some of the highest publication rates. Beyond simple conversion rates, the Netherlands also performed strongly on impact metrics, having the highest percentage of articles in high-impact journals (40.0%) and the second-highest median JIF (4.6). In contrast, the United States showed a more moderate publication rate (39.7%) but achieved the highest median citations per year (5.2). The persistent high publication rates from certain European countries could be related to their well-established research ecosystems and institutional experience in navigating the process from conference presentation to peer-reviewed publication. However, a major shift is evident in the volume of abstract submissions. While Germany and Italy were the countries that presented the most abstracts in past meetings, our study shows a notable change, with China now presenting the second-highest number of abstracts (*n* = 281) after Italy [3, 7, 9]. Furthermore, the publication output and success of Asian countries have shown notable growth, with South Korea achieving one of the highest publication rates (62.2%). This trend may reflect the increasing globalization of the congress and the expanding scientific output from research centers in Asia. When analyzed by continent, abstracts from Asia achieved a significantly higher publication rate than those from Europe (52.3% vs. 43.6%). This finding aligns with broader trends in radiological research [24]. Previous ECR analyses have also shown a steady increase in contributions from Asian countries over the past two decades [3]. Among Asian countries, South Korea achieved one of the highest publication rates in our study (62.2%). In a separate study, South Korea has been reported to rank second globally in academic quotient (publications per radiologist), which authors attributed to substantial investments in radiology resources and imaging technology [25, 26]. Selection bias may also contribute to this pattern, as geographic distance and travel costs may lead Asian institutions to preferentially present their strongest, most publication-ready research at European congresses. However, substantial within-region heterogeneity was observed. Among Asian countries, South Korea achieved a 62.2% publication rate while India reached 20.3%. This variability suggests that publication success may be influenced by multiple institutional and national factors beyond geographic region alone.

Articles derived from earlier ECR studies were most frequently published in *European Radiology* [7, 15]. In contrast, RSNA (Radiological Society of North America) abstracts were predominantly published in *Radiology*, indicating that regional differences may influence the leading destination journals [16]. In our cohort, publications appeared in 254 journals, with *European Radiology* being the most frequent. This higher number compared with earlier reports may reflect both the expansion of PubMed/MEDLINE-indexed radiology journals and the increased number of abstracts presented at ECR (from ~1000 in 2000–2010 to ~1800 in 2019) [3, 15].

Analyses of ECR 2010 showed that although international collaborations had slightly higher publication rates, no significant differences were found across local, national, and international groups [3]. In our study, no significant differences were observed either, but the share of abstracts arising from national and international collaborations increased compared with 2010, possibly reflecting the congress’s growing international participation and the rise of multicenter studies. This trend is particularly valuable, as multicenter collaborations often lead to larger sample sizes and more generalizable research findings.

Regarding study design, earlier literature has generally reported higher publication success for prospective studies [1, 11, 27–29]. In the ECR 2010 analysis, while prospective and retrospective abstracts showed comparable overall publication rates, prospectively designed studies were more commonly published in journals with higher impact factors [3]. Conversely, an evaluation of abstracts from the European Society of Gastrointestinal and Abdominal Radiology (ESGAR) meetings found that retrospective studies achieved significantly higher publication rates than prospective ones [11]. In our study, no significant difference was observed between prospective (47.4%) and retrospective (46.5%) designs. However, the proportion of prospective abstracts decreased compared with the previous meeting. This decline in prospective abstracts compared with earlier data may reflect shifting research practices and priorities in radiology. Nevertheless, both prospective and retrospective designs continue to contribute substantially to the field.

Analyses of ECR 2010 found no significant differences across imaging modalities, though PET-CT, MRI, and CT showed higher publication rates, while ultrasound ranked lower [3]. In our study, MRI (52.1%), ultrasound (51.1%), and PET-CT (46.7%) demonstrated the highest publication rates. Mammography abstracts showed a more modest publication rate (39.5%), but when published, they tended to appear in journals with higher JIF values. PET-CT abstracts achieved relatively high publication rates and, when published, tended to appear in journals with higher JIF values and citation counts. This pattern may reflect the clinical utility and broad applicability of hybrid imaging techniques.

The high publication rate for ultrasound is consistent with some previous studies of national radiology congresses that reported similar findings [30]. The notable increase in its publication rate at ECR, however, could be attributed to recent advancements in the field, such as the growing investigation of techniques like contrast-enhanced ultrasound and elastography, which have opened new avenues for clinical research [31–33]. The differences observed in our study, absent in 2010, may therefore reflect technological progress creating more opportunities for high-impact research in specific modalities. However, we believe that factors independent of the imaging modality, such as the study’s novelty, methodological quality, and sample size, are likely the primary determinants of an abstract’s eventual publication success.

Previous analyses of ECR 2010 reported significant differences across subspecialties, with Gastrointestinal imaging being the most frequently presented. However, the subspecialties with the highest publication rates varied even between two independent studies analyzing presentations from the ECR 2010 congress; one study cited Pediatrics and Thoracic imaging as having the highest rates, while the other identified Molecular imaging and Cardiac studies as having the highest rates [3, 7]. This discrepancy likely stems from methodological differences in how subspecialties were defined and categorized across those two studies. In both ECR 2000 and 2010, the ‘Radiographers’ category consistently showed low publication rates [7, 9].

Our findings from ECR 2019 show both consistencies with and deviations from previous trends. In our study, Neuroradiology was the most frequently presented subspecialty. Consistent with previous congresses, the ‘Radiographers’ category again demonstrated a low publication rate. This may reflect the nature of research in this subspecialty, which often emphasizes practice, workflow, and educational topics rather than clinical diagnostic studies. Furthermore, the publication trend for pediatric radiology appeared to fluctuate, as the high publication rate (63%) reported in 2010 was not sustained in our study (38.4%). Both Gastrointestinal (63.3%) and Abdominal radiology (61.8%) demonstrated high publication rates, marking a notable increase for Gastrointestinal imaging compared to the lower rates reported in 2010 [3].

At ECR 2019, Breast imaging achieved the highest median JIF (4.9) and the highest proportion of publications in high-impact journals (45.6%). The AI/ML category, while showing a moderate publication rate (44.4%), demonstrated the highest median annual citation count (10.5), which may reflect the growing scientific interest in artificial intelligence applications in radiology.

More than half (53.5%) of the abstracts at ECR 2019 did not progress to full-text articles. Previous studies most often cite lack of time after the meeting as a barrier [34, 35]. For some, conference presentation is considered a sufficient endpoint, and a “lack of intention to publish” has also been reported [30]. Less commonly, co-authorship issues or negative results are involved [36]. Researchers may also be discouraged when findings appear uninteresting or fail to confirm expectations. Yet underreporting such results creates gaps and bias in the literature, threatening scientific progress. Addressing these barriers by encouraging the dissemination of all findings, supporting authorship practices, and providing institutional incentives may help reduce the publication gap in future congresses.

This study has several limitations. First, our literature search was restricted to PubMed/MEDLINE to ensure methodological consistency and comparability with previous studies. While MEDLINE indexing indicates a certain level of quality, articles in lower-visibility or non-indexed journals may have been overlooked. In addition, language barriers could have limited the inclusion of non-English publications, potentially underrepresenting research from some countries. Publication outcomes may also be influenced by editorial and regional publication dynamics, such as journal selection preferences or differences in acceptance policies across regions. Second, citation counts were collected using Google Scholar for its broad coverage and accessibility, but it also includes non-peer-reviewed sources such as theses, proceedings, and preprints, which may have inflated citation numbers. Third, because the ECR 2019 Book of Abstracts lacked detailed institutional affiliations, collaboration was classified solely by geographic location, which may not fully capture partnerships within the same city. Furthermore, abstract-to-publication matching was manual and, despite a systematic approach, may carry a margin of error. In addition, although not directly analyzed, the COVID-19 pandemic may have influenced publication rates in certain subspecialties, particularly chest and emergency radiology, where clinical workload and research priorities were temporarily shifted. We did not perform post-hoc pairwise comparisons due to multiple testing concerns. Results from categories with relatively small sample sizes should be interpreted with caution, as they may have limited statistical power. Finally, our study compares two discrete time points (2010 and 2019), so observed trends may reflect period-specific rather than continuous variation. Future research, including data from additional congress years, will be required to validate these patterns. Despite these limitations, our study provides novel insights by directly comparing publication outcomes over nearly a decade and offers an updated perspective on the evolution of research dissemination at ECR.

## Conclusion

In this study, nearly half of the oral abstracts presented at ECR 2019 were subsequently published as full-text articles indexed in PubMed/MEDLINE, showing a modest but non-significant increase compared with ECR 2010. The landscape of contributors has become increasingly global, with a marked rise in participation from Asian countries. Publication outcomes varied substantially across countries, imaging modalities, and subspecialties. Breast imaging and AI/ML showed high journal impact and citation metrics. These findings reflect contemporary trends in radiological research. Continuous monitoring of publication patterns across multiple years, while considering database limitations and regional variations, will be essential to better understand the factors that facilitate or hinder the successful translation of conference presentations into peer-reviewed scientific output.

## Data Availability

The datasets used and/or analyzed during the current study are available from the corresponding author on reasonable request.

## Abbreviations

AI/ML: Artificial intelligence/machine learning
ECR: European Congress of Radiology
GI: Gastrointestinal
IQR: Interquartile range
JIF: Journal impact factor
